# Historical Zoonoses and Other Changes in Host Tropism of *Staphylococcus aureus*, Identified by Phylogenetic Analysis of a Population Dataset

**DOI:** 10.1371/journal.pone.0062369

**Published:** 2013-05-07

**Authors:** Marcus A. Shepheard, Vicki M. Fleming, Thomas R. Connor, Jukka Corander, Edward J. Feil, Christophe Fraser, William P. Hanage

**Affiliations:** 1 MRC Centre for Outbreak Analysis and Modelling, Department of Infectious Disease Epidemiology, School of Public Health, Imperial College London, London, United Kingdom; 2 Department of Biology and Biochemistry, University of Bath, Claverton Down, Bath, United Kingdom; 3 Wellcome Trust Sanger Institute, Hinxton, Cambridgshire, United Kingdom; 4 Department of Mathematics and Statistics, University of Helsinki, Helsinki, Finland; 5 Center for Communicable Disease Dynamics, Department of Epidemiology, Harvard School of Public Health, Cambridge, Massachusetts, United States of America; Columbia University, United States of America

## Abstract

**Background:**

*Staphylococcus aureus* exhibits tropisms to many distinct animal hosts. While spillover events can occur wherever there is an interface between host species, changes in host tropism only occur with the establishment of sustained transmission in the new host species, leading to clonal expansion. Although the genomic variation underpinning adaptation in *S. aureus* genotypes infecting bovids and poultry has been well characterized the frequency of switches from one host to another remains obscure. We sought to identify sustained switches in host tropism in the *S. aureus* population, both anthroponotic and zoonotic, and their distribution over the species phylogeny.

**Methodologies/Results:**

We have used a sample of 3042 isolates, representing 696 distinct MLST genotypes, from a well-established database (www.mlst.net). Using an empirical parsimony approach (AdaptML) we have investigated the distribution of switches in host association between both human and non-human (henceforth referred to as animal) hosts. We reconstructed a credible description of past events in the form of a phylogenetic tree; the nodes and leaves of which are statistically associated with either human or animal habitats, estimated from extant host-association and the degree of sequence divergence between genotypes. We identified 15 likely historical switching events; 13 anthroponoses and two zoonoses. Importantly, we identified two human-associated clade candidates (CC25 and CC59) that have arisen from animal-associated ancestors; this demonstrates that a human-specific lineage can emerge from an animal host. We also highlight novel rabbit-associated genotypes arising from a human ancestor.

**Conclusions:**

*S. aureus* is an organism with the capacity to switch into and adapt to novel hosts, even after long periods of isolation in a single host species. Based on this evidence, animal-adapted *S. aureus* lineages exhibiting resistance to antibiotics must be considered a major threat to public health, as they can adapt to the human population.

## Introduction

*Staphylococcus aureus* is a commensal and pathogen of both humans and many animal species [1]–[3]. Distinct lineages have been identified within the global population of *S. aureus* that associate closely with specific hosts [2]–[6]. While the mechanism of adaptation to host environments is not fully understood, it has been shown that such host tropisms are associated with adaptive evolution, in particular at immunologically relevant genes such as those encoding proteins determining virulence and cell-adhesion [4], [7]–[9]. However, adaptation to one host species does not prevent occasional infection of other species [5], [10]–[14]. The majority of cases where genotypes have been isolated outside of their typical host species probably represent spillover events; transient infections from one host species to another which do not last long, and die out without establishing transmission within the new host population [13]–[15]. These are distinct from rarer interspecies transmission events that lead to sustained transmission and establishment within the new host species [6], [16]. Only a small number of such genuine host-switching events have been studied and documented [5]–[7], [16], despite the broad distribution of *S. aureus* genotypes across host species [4], [7], [16]. These switches are uniformly anthroponotic; to our knowledge, there have been no documented cases of deep lineages of human adapted *S.aureus* originating from animal adapted strains in the past and having lost their animal association (this is distinct from infections involving single genotypes such as ST398, which are well known and are difficult to distinguish from spillover events). The total number of occasions where lineages have adapted to transmission within a novel host species remains uncertain but such information is vital to gauging the risks posed by zoonotic infection.

Zoonotic transfer of bacterial pathogens, either through contact or the food chain, represents a serious threat to public health. In particular, potentially zoonotic pathogens that display resistance to antimicrobials used in humans, such as *S. aureus,* and *Escherichia coli* are a matter of serious concern [17]–[20]. It has been shown that one lineage of bovine staphylococci is hypersusceptible to the acquisition of vancomycin resistance from enterococci. The risk posed by Vancomycin-Resistant *Staphylococcus aureus* (VRSA) [20] illustrates the need to understand the dynamics of interspecies transmission. The volume of antibiotics used in agriculture is greater than in human medicine, even in countries where antibiotics are well controlled [21]. The intimate linkage between human and veterinary medicine has been recognised for some time, and organisations such as the OneHealth Initiative aim to promote a unified approach to the practise of healthcare. Methicillin-Resistant *Staphylococcus aureus* (MRSA) is common in animals, and recently a commonly resistant sequence type (ST) ST398 has drawn attention due to its high transmissibility between livestock and humans [22], [23]. It is considered to be a novel zoonotic, and is an emerging threat to public health.

The *S. aureus* genotypes that are known commensals or pathogens of specific animal species are thought to originate from anthroponoses, that is to say they have arisen from *S. aureus* colonising humans [5]–[7], [16]. Despite the interest in ST398, and current zoonotic dynamics, the possibility of historical zoonotic transfers in the opposite direction, i.e. from animals to humans, has attracted less attention. The evidence for historical population dynamics is preserved within the population phylogeny. Genetic data, together with ancillary information on the host species from which genotypes were isolated, are available from large online databases collected for epidemiological purposes. Transitions between host species can be identified as switches between habitats, leading to a change in the context from which genotypes are isolated, occurring at particular points in the population phylogeny. Here we analyse a database for *S. aureus* in order to identify host switching events, and in particular to assess whether historical zoonoses can be detected.

## Results

We analysed sequences from the Multi-Locus Sequence Typing (MLST) database for *S. aureus* using the program AdaptML; using as input a maximum likelihood phylogeny generated from the concatenated MLST loci of 696 distinct genotypes, and metadata on host species obtained from www.mlst.net. AdaptML groups sequences associated with ecological distributions called ‘habitats’ which are inferred from associated metadata by a hidden Markov model (HMM). These habitats represent distinct groups of genotypes with shared ecology and are hypothesized to be low-dimensional projections of the realised niche of the isolates under study. In our study we have the niche projected onto a single axis, host type. It is important to note that in the way that a niche is characterised by a particular distribution of genotypes with respect to all environmental covariates, so the habitats inferred by AdaptML represent distributions of genotypes with respect to the specific environmental covariates under consideration. This permits a degree of flexibility within our inference as genotypes are not assumed to be constrained to one or either habitat, which reflects the nature of interspecies transmission in a species such as *S. aureus*. Proportions of genotypes are found out of their expected habitats, many due to spillover events, but a few due to infrequent host switching events [14], [24].

**Figure 1:**
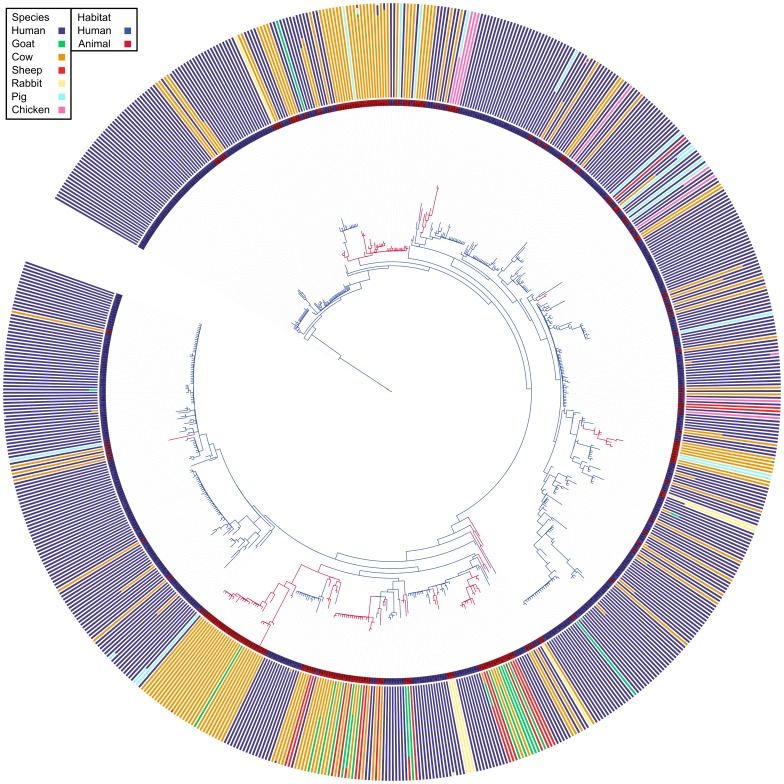
A maximum likelihood phylogeny of 696 MLST STs derived from human and animal hosts. A maximum likelihood phylogeny of 696 MLST STs derived from human and animal hosts. Branch colours describe habitat associations inferred by AdaptML (Human – Blue, Animal – Red). External bars represent the normalised proportions of isolates from each of the seven species for each ST. Internal colours represent the most common source of isolation for each ST, this is used as the ecology marker in the AdaptML inputs. (Human – Blue, Animal – Red).

AdaptML inferred two habitats, one containing genotypes predominantly isolated from humans, and the other genotypes mainly isolated from animals. More precisely the distribution of human and animal genotypes across the inferred habitats is described by a set of emission probabilities. These are probabilities that a genotype is found in a specific habitat (in this case human or animal), given that it was isolated from either a human or animal source. The emission probabilities for the two habitats found within this dataset are listed in table 1. These habitats and the underlying data are described in figure 1. It should be noted that genotypes can be isolated outside of their normal host species, these represent spillover transmissions [14]. This is reflected in the isolate distribution bar charts attached to each ST in figure 1.

**Table 1:** The emission probabilities for two inferred habitats.

Habitat	Human	Animal
**H**	0.885	0.115
**A**	0.143	0.857

Habitat H is predicted to be the predominantly human habitat, habitat A is predicted to be predominantly animal.

**Figure 2:**
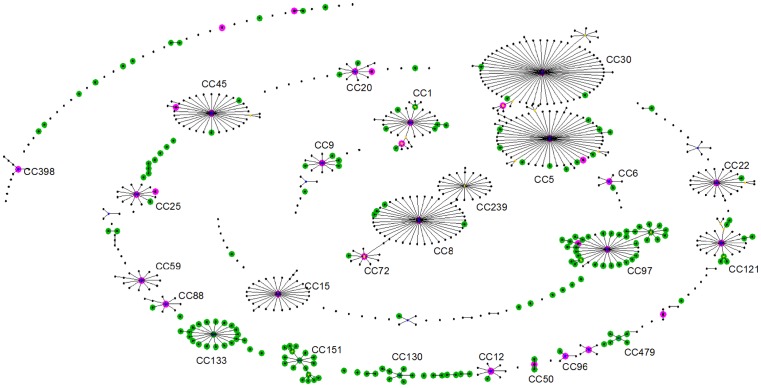
A Comparative eBURST plot of 696 STs from human and animal hosts. Comparative eBURST of 696 STs. Genotypes from the human habitat are shown in black, genotypes from the animal habitat are shown in green. Genotypes found in both habitats are highlighted in pink. Nodes with yellow centers indicate genotypes defined by eBURST as sub-founders within a larger clonal complex.

Previous work has used the eBURST approach to identify clonal complexes (CCs) of closely related genotypes associated with animal populations, and we have examined the position of these in our analysis; a representation of all the clonal complexes in the population sample is presented in figure 2. Where a clade contains genotypes of a recognised CC we refer to it as such, to aid consistency of nomenclature with previous work. These CCs represent distinct lineages, which share ecological traits as a result of common descent; the AdaptML-inferred habitat association of each clade allowed us to label each clade and its corresponding CC as either human or animal. Examining the tree and inferred habitats shown in figure 1, we observe twenty points where the habitat association of two vertically adjacent nodes is different. According to the assigned habitats of the basal and distal nodes of the branch joining these nodes, we can infer the type of host-switch which has occurred. To maximise the ability of the AdaptML analysis to detect transitions from human adapted genotypes (that form the vast majority of the dataset) we have pooled non-human sources into a single ‘animal’ type. The type used for analysis is highlighted on the internal ring of figure 1, red bars for animals, blue for humans. The exterior bars of figure 1 highlight the extent of variation in host species for each genotype. In this figure there are 17 visible anthroponoses. Six of these have been identified previously, and describe the largest known clades of animal-associated *S. aureus*: CC97, CC126, CC130, CC133, CC151 (occasionally described as CC705), and CC385 [4], [6], [7], [16], [19], [25]–[27]. The remaining eleven anthroponoses are novel; they describe six single genotype switches, three clades of two genotypes and two clades of three genotypes. The small size of these clades may explain why they have previously gone unnoticed. Of these, two are novel anthroponoses into rabbit populations: ST414 and the clade composed of ST409, ST415, and ST416. The CC25 and CC59 clades, together with ST93 are apparently the result of historical zoonoses. CC25 and CC59 are comparable in terms of numbers of genotypes within each lineage, however the host distribution of isolates for both lineages differs. As listed in table S1, CC59 contains a single animal isolate to forty human isolates, whereas CC25 has a majority of animal isolates, although as shown by table S2 there is a clear majority of human genotypes. We note that 73 of the 78 cow isolates within CC25 come from a single study, possibly biasing the sample. If a single representative isolate is included from each study, the number of cow isolates for ST25 drops from 74 to 4, while the number of human isolates drops from 39 to 5. Table S3 shows how a correction for sampling shifts the clade towards a human consensus; going from a ratio of human to animal isolates of 50∶81 to a ratio of 16∶10.

Branch lengths are a crucial component of how AdaptML infers habitats, and this raises the possibility that our analysis might be compromised by recombination. Although it is known that the *S. aureus* undergoes recombination at a relatively low rate, we checked this by removing each gene in turn and constructing seven jackknife trees based on the remaining six genes (Figures S3, S4, S5, S6, S7, S8, S9) [28]–[30]. By considering the resultant pairwise genetic distances and similarity in regions of the phylogeny where switching had been inferred, we were able to determine the likelihood that recombination had confounded our results. Whilst the overall similarities of the topologies of the jackknife trees to each other, and to the main tree were typically low (table S4), analysis of the jackknife trees using AdaptML reconstructed the majority of the switches seen in the main tree. The jackknife trees tended to overstate the number of zoonoses, and understate the number of anthroponoses, with respect to the seven gene tree (table S5).

**Figure 3:**
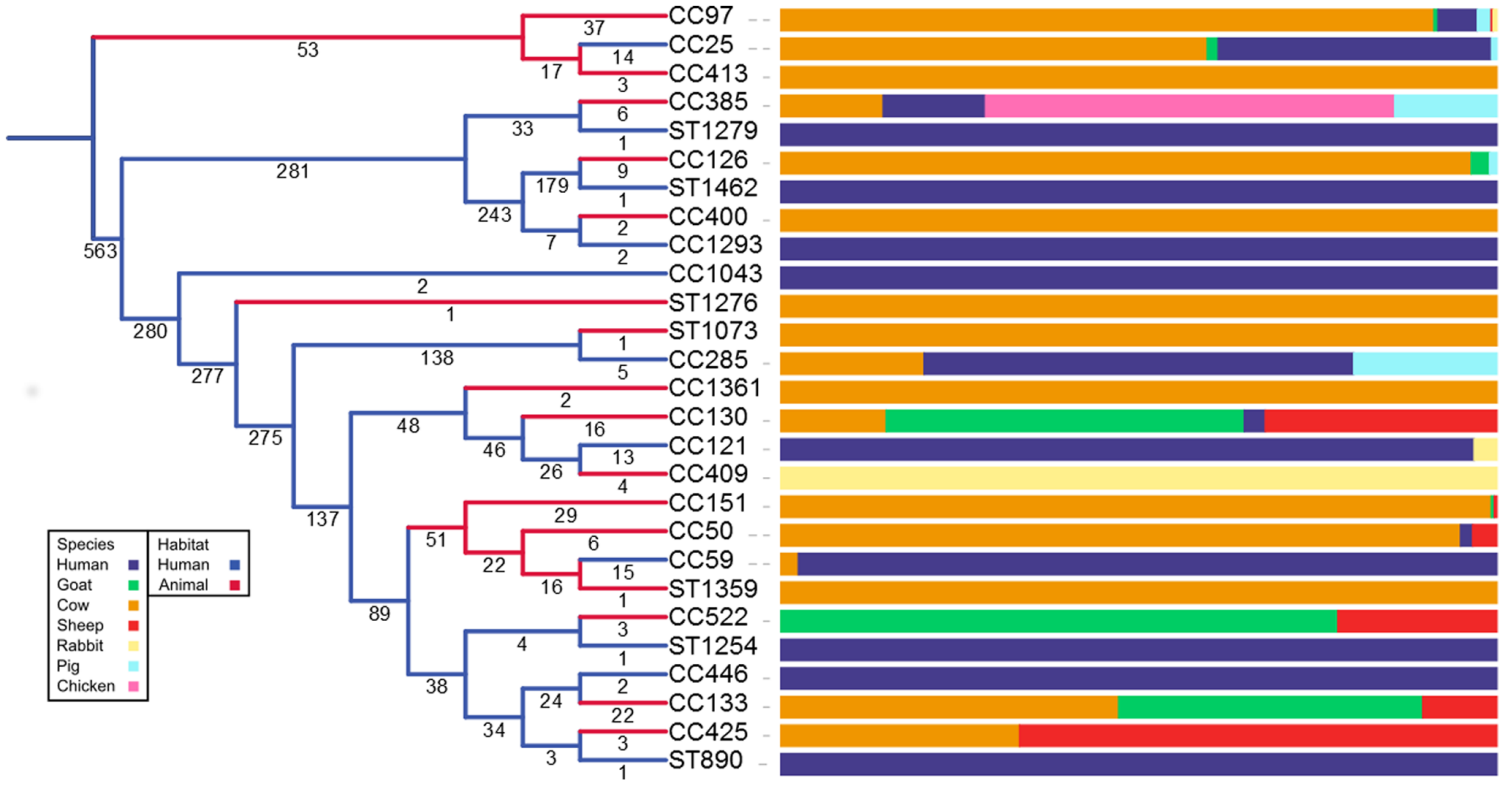
A pruned cladogram describing all 15 host switching events within the *S. aureus* population sample. Values attached to branches represent the number of unique STs entering each node, from left to right. Leaf labels indicate the defining ST or CC. Bars represent the distributions of isolates for each terminal clade, colours for each species are the same as in figure 1. Clades which underwent no switches are pruned from the tree at nodes where the branch containing them is uniform with the ancestral state. Branch colours represent the inferred habitat type (blue – human, red – animal). Asterisks mark points where host switching events occur.

Having examined every switch we eliminated five of the switches found in the original analysis as recombinational artefacts; the structure and relationships of the 15 remaining host switches are described using a pruned cladogram in figure 3. These switches (STs 93, 136, 411, 414, and 1119) are all single ST switches, and appear have been placed outside of their true clade due to recombination. Of these switches, only ST93 saw a change in inferred nature, changing from a predicted zoonosis to a component of an animal clade. All the anthroponoses merged into other anthroponotic switches. Although the larger CC130, CC151, and CC385 clades showed evidence of internal recombination this had no clear effect on their status as switches. Similarly, despite some evidence of recombination separating CC59 and CC151 this did not affect the inference of a switch.

It is noteworthy that the true location of CC398 within the phylogeny seems to be adjacent to the CC50, CC59 and CC151 clades; as a result of one or more recombinations it is located elsewhere in the main tree shown in Figure 1. Within the jackknife trees CC398 is inferred as a human clade, as in figure 1, and it is adjacent to CC59 in three trees: -aroE, -gmk_ and –yqiL; in the –aroE jackknife tree the position of CC398 disrupts the prediction of a zoonotic switch at the base of CC59, and splits CC50 and CC151 apart into two separate switches. This change results from the inference of human a human habitat one branch higher in the fraction of the phylogeny ancestral to all of the CCs 50, 59, 151, and 398. In each case a new clade composed of 10 STs is inserted into this region of the tree (STs: 291, 398, 580, 601, 804, 813, 1006, 1033, 1450, and 1646); when the pairwise Hamming distances are calculated for the 10 STs of the CC398 clade, and the STs of the most adjacent clade (CC59 in the –qroE tree, STs 687 and 689 in the –gmk_ tree, and ST1526 in the –yqiL tree) the bulk of the sequence distance is concentrated in the aroE gene. In all cases between 25% and 52% of the total distance of any pair is accounted for by this single gene. No obvious donor presents itself from an analysis of the ST profiles of these STs in a comparison with each other, and the MLST database as a whole, and the movement in three trees makes it hard to identify a single recombination event that may have separated CC398 from CC59 and its relatives. The significance of these movements remains unclear, and in the –gmk_ and –yqiL jackknife trees both CC59 and CC398 are predicted to be human clades arising from zoonosis by AdaptML.

By dating the age of nodes which straddle a habitat transition within the tree we provide estimates of date ranges where host switches occurred. We have estimated the date range encompassing the zoonotic switches that lead to the CC25 and CC59 lineages using BEAST; estimates for these dates are presented in table S6. In both cases the habitat transition is estimated to have taken place centuries ago, supporting a previous study [6].

## Discussion

Our analysis of *S. aureus* isolates in the MLST database reveals 20 host switches over time, of these, 15 have been shown to be robust to recombination. These switches are predominantly from a human source to animal populations, but they include two cases where it appears genotypes adapted to transmission in animal populations have moved back to a lifestyle based on predominantly human to human transmission. This result supports the view that human and veterinary medicine are intertwined [31], and that the risk of animal adapted genotypes becoming adapted to transmission in humans must be taken seriously. However, we note that such zoonotic events are likely to be rare, with the most recent occurring several centuries ago.

We have identified two previously unknown zoonoses, resulting in lineages dominated by isolates from human sources that arise from the midst of animal-associated clades: CC25, and CC59. The phylogenetic neighbours of both clades are unambiguously animal (CCs 97 and 151 represent the most extensive and best characterised groups of *S. aureus* genotypes associated with animal hosts [4], [7]). These zoonoses are thus defined with high confidence. Given the branch lengths and the apparent sizes of these clonal expansions we conclude that these zoonoses are not recent, and have had time to differentiate within human hosts. Given the size of the CC25 and CC59 clades, and their relative distance from adjacent clonal complexes, we infer that they are long established in the human population. Our dating analysis (Table S6) supports this, suggesting that they have been in existence for centuries at the very least. It should be noted that our dating approach makes use of an independent estimate of clock rate from recently diverged genomes [32]. It is possible that over a longer time scale, a slower clock rate should be applied [33]. If this is the case, the estimates in table S6 should be pushed even further back in to the past. We therefore conclude that there is scant evidence for zoonoses occurring in the last 500 years. However, this divergence date is an upper bound estimate as there may be animal associated genotypes which are more closely related to the human adapted strains, but which have yet to be sampled. In contrast, host switches in the opposite direction (human to animal) seem to have occurred far more recently [3], [7], [9], [16]. The naïve parsimony interpretation of this is that humans are the basal host species of *S. aureus*, or at least they are the prime interspecies vector; however without deeper sampling of certain geographical regions and wild animal species it is impossible to draw a solid conclusion.

There is good evidence for 13 transitions from humans to animals within the population phylogeny, including six that have previously been described and which support the ability of our approach to detect host switching (CC97, CC126, CC130, CC133, CC151, and CC385 [4], [7], [16], [19], [25]–[27]). The underlying species composition detailed in the exterior bars of figures 1 and 3 show that the host composition of clades varies, and are generally heterogeneous in their relative compositions. Intuitively this is unsurprising; the category ‘animals’ contains multiple species, and so we would not expect it to exhibit less within-class immunological variation than the variation between animals and humans; thus the contrast is somewhat artificial and warrants further study with an enriched dataset for non-human isolates. If anything we might expect a greater adaptive step between a mammalian host and an avian host than between different mammal hosts. There is also the matter of transmission - we expect the degree of within-species association to greatly outstrip any interspecies association for almost all animals. This may have been different in the past, when there were a greater proportion of mixed smallholdings, but not so in modern agriculture, and it is reasonable to suggest that crowded populations of single host species facilitate adaptation to those species, both through increased contact rates, and the negative effects of farming intensity on the immune systems of farmed animals.

The other seven clades arising from anthroponoses contain relatively small numbers of genotypes, but it is notable that two involve apparent independent switches to transmission in rabbit populations; these are well supported.

MLST uses only seven loci, which limits our ability to detect recent switches. This is shown by the failure of our analysis to identify ST398 as animal-associated, despite excellent epidemiological evidence for a livestock association [22], [23], [34]. This is explained by an examination of ST398’s native clade, shown in detail in figure S1. When constructing the habitats across the tree the analysis considers both the branch length of each genotype and the assigned types of related genotypes. This helps to avoid identification of false switches because a switched genotype must be sufficiently established within its new host population to have undergone diversification. ST398 is located at the end of a relatively short branch, and resides in a well-formed clade of genotypes that are entirely derived from human sources. As shown in figure S1: within CC398, almost all other closely related genotypes are recovered from human sources; in total only two of the 50 most closely-related sequence types have a majority of animal isolates (STs 122 and 1120) and 45 show only human isolates. Were ST398 a long established animal-associated genotype we would expect to observe a set of closely related, predominantly animal-associated genotypes in the immediate phylogenetic neighbourhood. Our analysis of recombination within the *S. aureus* phylogeny suggests that the true neighbours of CC398 may be the human to animal switching clades CC50 and 151, and if this relationship can be substantiated then it might suggest a similarity in zoonotic tendency. However, even in the jackknife trees where the CC398 clade was seen to move proximal to these clade, the internal host distribution of CC398 remained unchanged, it was still a strongly human-associated clade(figures S4, S6, and S9). It has been noted elsewhere that ST398 has a complex epidemiology, an observation with which we concur; in particular recent work has highlighted the differences in virulence and transmissibility between human and animal clones of ST398 [9], [35]–[37]. Taken together with the comments on dating habitat switches above, our results are consistent with ST398 having undergone a host transition from humans to animals extremely recently, which agrees with more detailed studies of this genotype [9]. This is a cause for concern, as lineages which pose the greatest risk for switching are thought to be generalists that infect multiple species [38]. Whether the epidemiology of ST398 presages a wider shift in the CC398 lineage, or whether it is a singular event that will found a novel animal-associated lineage from the recent human-associated ancestor remains to be seen. The temporal proximity to the point of host switching offers a credible explanation for the persistent affinity for human hosts shown by isolates of ST398; this observation is supported by whole-genome analyses [9]. Such a view is further supported by a study of *S. aureus* isolated from healthy children in China, which showed that 8% of the carriage population corresponded to ST398; although these carriage isolates were MSSA and livestock-associated ST398 is typically MRSA. Although this study was restricted to a single city (Chengdu), it raises the possibility that this genotype might be commonly carried asymptomatically amongst human populations, particularly in China, where the relevant data remains scarce [39], [40].

Shortcomings of the present work can be divided into those arising from features of the source data, and the analysis. In terms of the source data the MLST databases do not contain systematically collected surveillance data and hence may contain biases toward certain heavily studied populations. To mitigate against this we have expanded the core dataset with results culled from papers looking at animal hosts; furthermore, the core dataset has been heavily curated to remove spurious or anomalous results. As the algorithm does not consider the depth of sampling of individual sequences, oversampling of individual genotypes is not a major issue. However there is no clear way to correct for differential sampling of lineages in the data; to mitigate against this we imposed tighter cutoffs for the ascertainment of new habitats within AdaptML. When it comes to the analysis, the approach depends on a robust and accurate phylogeny. These phylogenies can be disrupted by recombination. Recombination between lineages found in different habitats may result in genotypes and lineages appearing to rise from an incorrect ancestral background. It should be noted however that the relative rate of recombination in *S. aureus* is relatively low, and is not expected to greatly impact the phylogeny [30]. In our analysis while recombination could be found in around half of all switching clades, only five switches were affected, and these were all single ST switches. Furthermore, the bootstrap values for the main tree give some estimate of the confidence we can have in the phylogeny, these are listed - for the branches where the historical host switches are inferred to have occurred - in table S7, and detailed further in figure S2. Hence we are confident in the majority of our identified switches, and welcome further research to examine the accuracy of our results.

### Conclusions

It is clear that genotypes of *S. aureus* commonly infect species that are not their native host. Between lineages there is evidence of preferential association and adaptation which dispels any notion that animals (including humans) represent a uniform host landscape to the pathogen. However, there have been several occasions where a genotype has switched host and founded a novel lineage. The findings presented here suggest that major host switches are infrequent, but spillover events are common and represent frequent opportunities for a genotype to become established in an additional host. This supports the concept that human and veterinary medicine should treated as a strongly linked fields, both clinically and epidemiologically, particularly with respect to practises such as the use of antibiotics.

## Materials and Methods

### Sources of Data

The data for this study were drawn from the publicly available database of MLST isolates, including a set of genotypes isolated from six types of animal (Cow, Sheep, Goat, Rabbit, Pig and Poultry) that were sequenced as part of the present study using the standard MLST protocol [41]. Characteristics of these additional genotypes are detailed in Table S8. For MLST, isolates are sequenced at seven housekeeping genes and each unique combination of alleles at the seven loci defines the allelic profile and the genotype. The concatenated sequence of all seven genes was used as input for AdaptML. Details of all isolates in the database were downloaded and the information was cleaned and consolidated into a set of 3042 isolates, for which there was reliable information regarding the species of isolation. The dataset was cleaned by a manual inspection of all 3042 entries, followed by consolidation and curation of metadata fields to yield an accurate and simplified dataset where a host species is identified for each isolate. Within the sample of 3042 isolates there are 696 distinct genotypes, with some genotypes having multiple representative isolates, while the majority of genotypes have only one. While twenty three species are represented in the database, we restricted the analysis to species found in association with at least ten different genotypes resulting in the following host species: humans, cows, sheep, goats, rabbits, pigs and chickens. We excluded genotypes associated with two highly divergent clades of *S. aureus*, one of which corresponds to the complex of genotypes including ST75 and ST1223 which has recently been identified as a putative novel Staphylococcal species [42]. We also excluded four genotypes which lacked complete sequence information (STs 159, 1166, 1463 and 1743). For each genotype we assigned a label indicating the host tropism based on the majority of isolates of that type in the database. In one case (ST912) we had equal numbers of human and animal isolates; however comparison runs using either possible host for ST912 showed no difference in the habitat distributions or emission probabilities. In the final figure ST912 is labelled as animal for the sake of convenience.

### AdaptML

In order to identify host associations within the global population sample we employed an empirical parsimony algorithm (AdaptML) [24]. The algorithm employs a HMM approach to group sequences into ecologically-similar habitats. The algorithm is agnostic with respect to the true number and distribution of habitats, and discovers true habitats by working down from a large number of randomly distributed habitats, to a smaller number of non-random habitats. During this process habitats which are correlated above a user-specified threshold are merged into each other. This is done to eliminate redundant habitats, and present only habitats which represent distinct ecologically-separated classes of genotypes. The AdaptML analysis results in a set of emission probabilities which describe the discrete habitats inferred within the tree [24].

Using AdaptML we reconstructed a credible description of past events taking as input a phylogenetic tree, the nodes and leaves of which AdaptML assigns to either human or animal habitats, estimated from extant host-association and the degree of sequence divergence between genotypes. In this study the predominant ecology under consideration was host tropism, specifically either human or animal. The method is entirely agnostic with respect to the distribution of true habitats and begins with nodes of the tree randomly distributed amongst an arbitrarily specified number of initial habitats (n = 10). Using a hidden Markov model nodes are moved between habitats in a probabilistic manner based on the structure of the phylogeny, in particular the branch lengths. The model parameters are then optimised by maximum-likelihood to find the best fit to the data. Having learned and optimised the model parameters, non-informative habitats are merged based on a user-specified parameter (0.01 for all analyses presented here). Merging is determined by testing the correlations of the emission probability distributions for all habitat pairs; where correlation exists above the specified threshold (0.99), the habitats are merged. Selection of this parameter exerts a degree of control over the number of habitats inferred in the final result; using the value chosen should offer a greater number of habitats, should many habitats be present.

### Phylogenetic Analysis

All trees used for the AdaptML analysis were produced using PhyML 2.44 [43] which generated suitable input files for AdaptML. jModelTest indicated that the GTR+I+Γ substitution model was the most appropriate for tree construction [44]. The detailed tree of the CC398 phylogenetic neighbourhood was produced using FastTree [45]. Tree figures were generated using the interactive Tree of Life web application (itol.embl.de) [46].

We determined dates for the major zoonotic host switching events using BEAST v1.6.1 [47]. We prepared sequence sets representing all genotypes common to the nodes at either end of a branch where a zoonotic switch occurs and estimated the age of the root for trees corresponding to each set. Using BEAUTi v1.5.4 we generated the necessary xml files; using a relaxed clock with an uncorrelated lognormal distribution and a substitution rate of 3.3×10^−6^ per site per year as suggested by Harris et al (2010) [32], [48]. For each node we performed eight runs of 50,000,000 steps, sampling every 100,000 steps; these were combined using LogCombiner v1.5.4. The treeModel.rootHeight prior was constrained to a uniform distribution in the range 0 to 10,000. All results were analysed using Tracer v1.5. For all runs the effective sample size was in excess of 8000.

## Supporting Information

Figure S1 **Detailed phylogeny of the phylogenetic neighbourhood of ST398.** An enlarged phylogeny of the clade surrounding ST398, showing the overwhelming tendency of isolates to be human-derived. Peripheral bars describe the proportions of isolates from different host types for each ST (Blue – Human, Orange – Cow, Light Blue – Pig).
(TIF)

Figure S2 **Bootstrapped maximum likelihood phylogeny of 696 MLST STs.** A maximum likelihood phylogeny of 696 MLST STs. Branch colours describe habitat associations inferred by AdaptML (Human – Blue, Animal – Red). Branches with bootstrap support of 80% or greater have their bootstrap values listed.
(TIF)

Figure S3 **A maximum likelihood phylogeny of 696 MLST STs built using 6/7 MLST genes, excluding** ***arc*** **.** A maximum likelihood phylogeny of 696 MLST STs derived from human and animal hosts. Branch colours describe habitat associations inferred by AdaptML (Human – Blue, Animal – Red). The colours of the tip labels describe the input host assignment for each sequence type, red for animal, blue for human. Tip labels coloured green represent STs that formed polytomies as a result of the *arcc* gene being excluded and were unparsed by the algorithm.
(TIF)

Figure S4 **A maximum likelihood phylogeny of 696 MLST STs built using 6/7 MLST genes, excluding** ***aroe*** **.** A maximum likelihood phylogeny of 696 MLST STs derived from human and animal hosts. Branch colours describe habitat associations inferred by AdaptML (Human – Blue, Animal – Red). The colours of the tip labels describe the input host assignment for each sequence type, red for animal, blue for human. Tip labels coloured green represent STs that formed polytomies as a result of the *aroe* gene being excluded and were unparsed by the algorithm.
(TIF)

Figure S5 **A maximum likelihood phylogeny of 696 MLST STs built using 6/7 MLST genes, excluding** ***glpf*** **.** A maximum likelihood phylogeny of 696 MLST STs derived from human and animal hosts. Branch colours describe habitat associations inferred by AdaptML (Human – Blue, Animal – Red). The colours of the tip labels describe the input host assignment for each sequence type, red for animal, blue for human. Tip labels coloured green represent STs that formed polytomies as a result of the *glpfc* gene being excluded and were unparsed by the algorithm.
(TIF)

Figure S6 **A maximum likelihood phylogeny of 696 MLST STs built using 6/7 MLST genes, excluding** ***gmk_*** **.** A maximum likelihood phylogeny of 696 MLST STs derived from human and animal hosts. Branch colours describe habitat associations inferred by AdaptML (Human – Blue, Animal – Red). The colours of the tip labels describe the input host assignment for each sequence type, red for animal, blue for human. Tip labels coloured green represent STs that formed polytomies as a result of the *gmk_* gene being excluded and were unparsed by the algorithm.
(TIF)

Figure S7 **A maximum likelihood phylogeny of 696 MLST STs built using 6/7 MLST genes, excluding** ***pta_*** **.** A maximum likelihood phylogeny of 696 MLST STs derived from human and animal hosts. Branch colours describe habitat associations inferred by AdaptML (Human – Blue, Animal – Red). The colours of the tip labels describe the input host assignment for each sequence type, red for animal, blue for human. Tip labels coloured green represent STs that formed polytomies as a result of the *pta_* gene being excluded and were unparsed by the algorithm.
(TIF)

Figure S8 **A maximum likelihood phylogeny of 696 MLST STs built using 6/7 MLST genes, excluding** ***tpi_*** **.** A maximum likelihood phylogeny of 696 MLST STs derived from human and animal hosts. Branch colours describe habitat associations inferred by AdaptML (Human – Blue, Animal – Red). The colours of the tip labels describe the input host assignment for each sequence type, red for animal, blue for human. Tip labels coloured green represent STs that formed polytomies as a result of the *tpi_* gene being excluded and were unparsed by the algorithm.
(TIF)

Figure S9 **A maximum likelihood phylogeny of 696 MLST STs built using 6/7 MLST genes, excluding** ***yqil*** **.** A maximum likelihood phylogeny of 696 MLST STs derived from human and animal hosts. Branch colours describe habitat associations inferred by AdaptML (Human – Blue, Animal – Red). The colours of the tip labels describe the input host assignment for each sequence type, red for animal, blue for human. Tip labels coloured green represent STs that formed polytomies as a result of the *yqil* gene being excluded and were unparsed by the algorithm.
(TIF)

Table S1 **Total numbers of isolates for each species, for all STs, within clades arising from a host switching event.** Total numbers of isolates for each species, for all STs, within clades arising from a host switching event; these are sorted into animal clades arising from a zoonosis and human clades arising from an anthroponosis.
(DOCX)

Table S2 **Table listing the total numbers of STs and isolates for each clade arising from a host-switching event.** STs and isolates are subdivided into those of either human (H) or animal (A) origin.
(DOCX)

Table S3 **List of isolates in the CC25 clade, showing correction for sampling.** This table lists the specific number of isolates from each host species for all STs found in the CC25 clade. The uncorrected numbers represent the totals as listed in the database, while the sample-corrected numbers represent the totals if only a single isolate of any given ST is included per species for each unique study listed in the database.
(DOCX)

Table S4 **Pairwise similarity between the topologies of all seven jackknife trees and the main seven-gene phylogeny.** Proportions of similarity between the topologies of the main tree (figure 1) and the jackknife trees built from 6/7 MLST genes (figures S3, S4, S5, S6, S7, S8, S9). Proportions represent the number of all nodes that are identical in both trees.
(DOCX)

Table S5 **Comparison of the number of inferred host switches in seven-gene, and six-gene jackknife phylogenies.** A list describing the basal habitat in both the main tree (figure 1) and the seven jackknife trees (figures S3, S4, S5, S6, S7, S8, S9), as well as the numbers of zoonoses and anthroponoses observed in each.
(DOCX)

Table S6 **Dates of zoonotic habitat transitions.** Dates of zoonotic habitat transitions in Figure 1, estimated using BEAST software with clock rate set to (3.3×10^−6^ substitutions per site per year). Date ranges represent the TMRCA (given by the treemodel.RootHeight parameter in BEAST) for the nodes at either end of a branch where the basal node is associated with the inferred animal habitat, and the distal node is associated with the inferred human habitat. Only the transitions giving rise to CC25 and CC59 had sufficient sequence information to generate a credible date.
(DOCX)

Table S7 **List of bootstrap values for branches where switches occur.** Bootstrap values of branches where host switches occur. Switches involving only a single ST are marked with an asterisk, and the bootstrap value of the immediate basal branch is given as a reference.
(DOCX)

Table S8 **List of supplemental isolates.** A table of supplementary animal isolates provided by EF which were included in the final dataset along with the isolates already present within the *S. aureus* MLST database.
(DOCX)
