# Supplementary figures and images for: Historical Zoonoses and Other Changes in Host Tropism of Staphylococcus aureus, Identified by Phylogenetic Analysis of a Population Dataset
Source: PLoS One. 2013 May 7;8(5):e62369. doi: 10.1371/journal.pone.0062369 (PMC3647051; doi:10.1371/journal.pone.0062369)

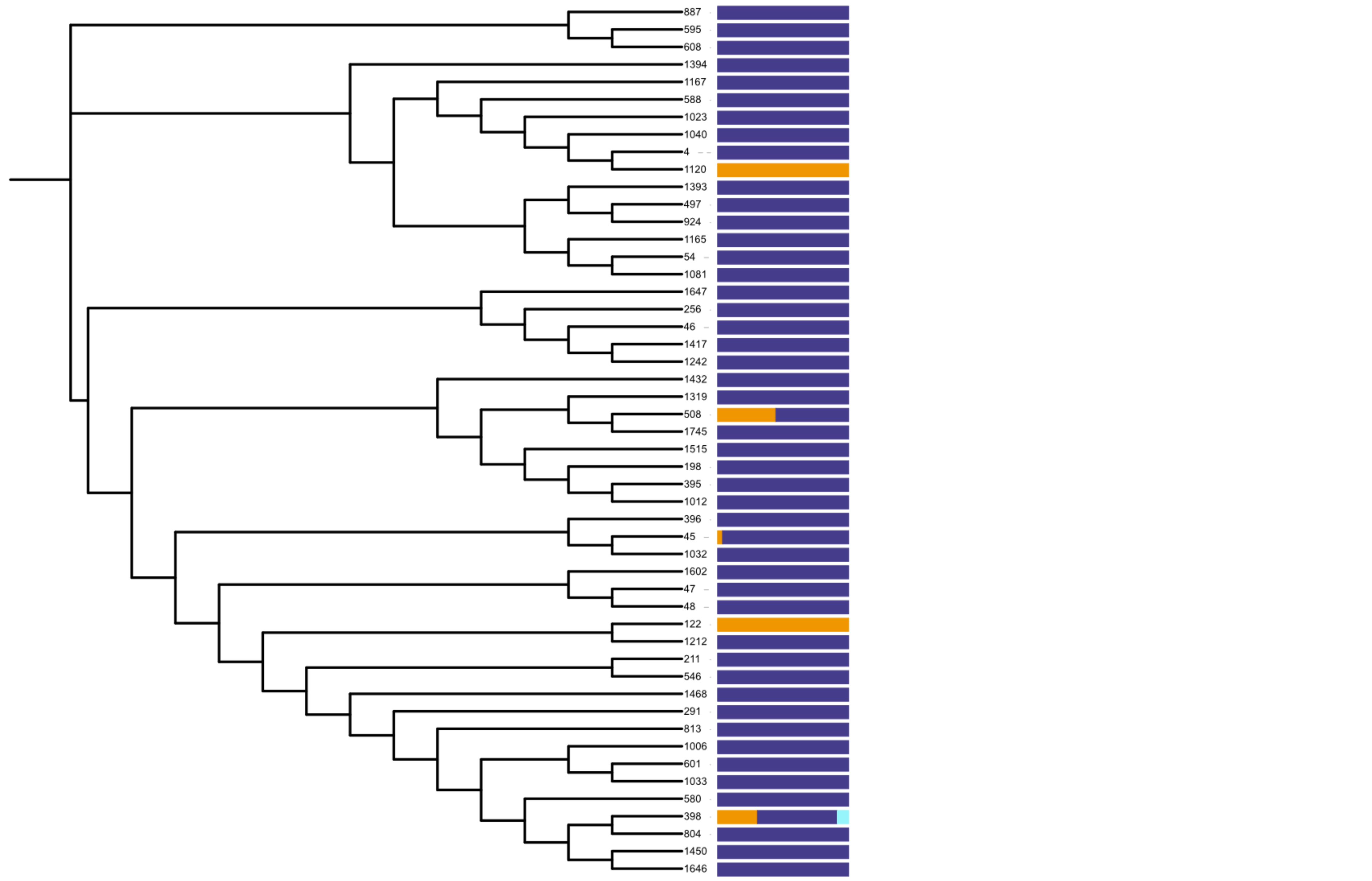

Supplement: Figure S1 — Detailed phylogeny of the phylogenetic neighbourhood of ST398. An enlarged phylogeny of the clade surrounding ST398, showing the overwhelming tendency of isolates to be human-derived. Peripheral bars describe the proportions of isolates from different host types for each ST (Blue – Human, Orange – Cow, Light Blue – Pig). (TIF) [file pone.0062369.s001.tif]

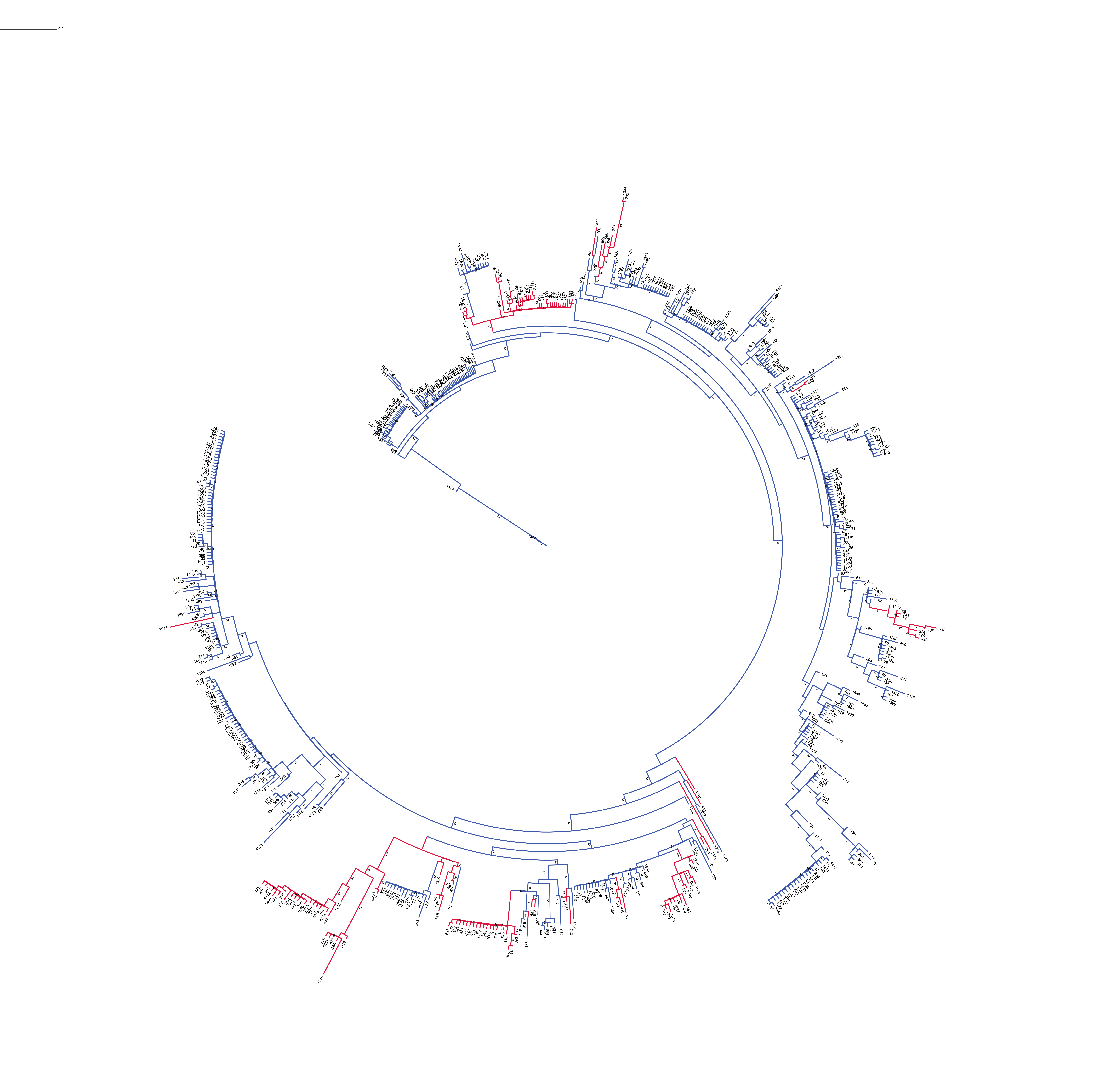

Supplement: Figure S2 — Bootstrapped maximum likelihood phylogeny of 696 MLST STs. A maximum likelihood phylogeny of 696 MLST STs. Branch colours describe habitat associations inferred by AdaptML (Human – Blue, Animal – Red). Branches with bootstrap support of 80% or greater have their bootstrap values listed. (TIF) [file pone.0062369.s002.tif]

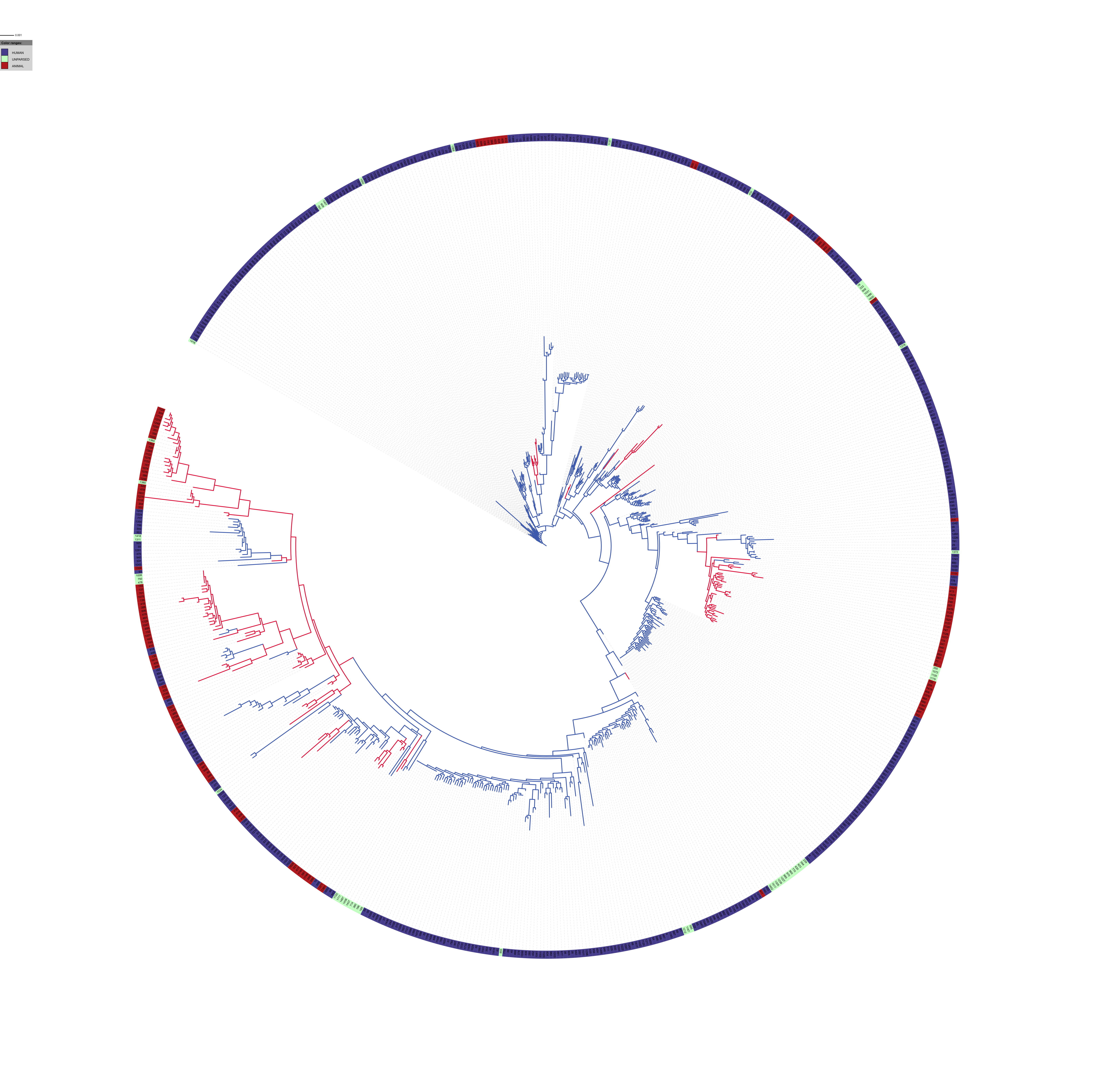

Supplement: Figure S3 — A maximum likelihood phylogeny of 696 MLST STs built using 6/7 MLST genes, excluding arc . A maximum likelihood phylogeny of 696 MLST STs derived from human and animal hosts. Branch colours describe habitat associations inferred by AdaptML (Human – Blue, Animal – Red). The colours of the tip labels describe the input host assignment for each sequence type, red for animal, blue for human. Tip labels coloured green represent STs that formed polytomies as a result of the arcc gene being excluded and were unparsed by the algorithm. (TIF) [file pone.0062369.s003.tif]

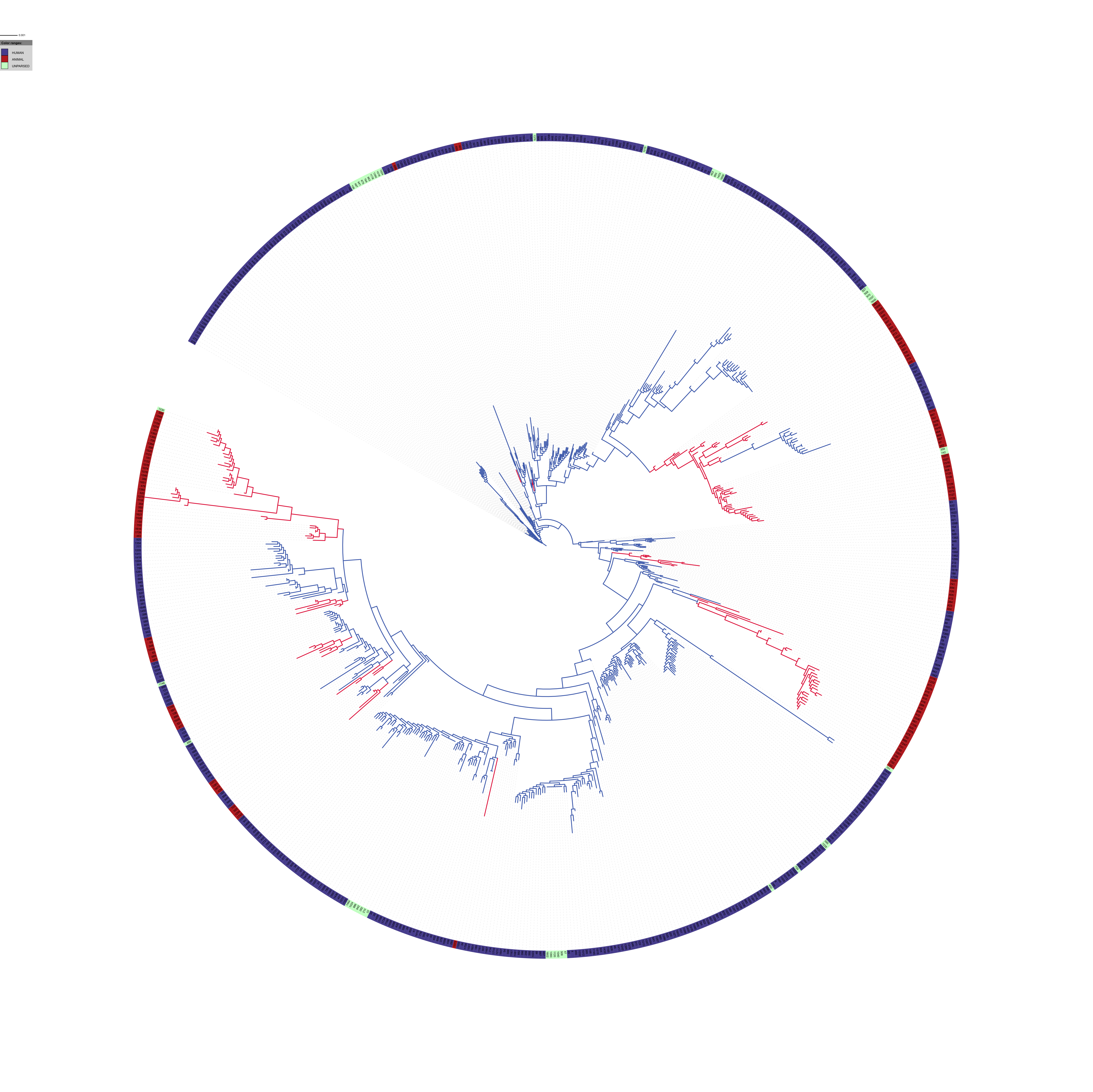

Supplement: Figure S4 — A maximum likelihood phylogeny of 696 MLST STs built using 6/7 MLST genes, excluding aroe . A maximum likelihood phylogeny of 696 MLST STs derived from human and animal hosts. Branch colours describe habitat associations inferred by AdaptML (Human – Blue, Animal – Red). The colours of the tip labels describe the input host assignment for each sequence type, red for animal, blue for human. Tip labels coloured green represent STs that formed polytomies as a result of the aroe gene being excluded and were unparsed by the algorithm. (TIF) [file pone.0062369.s004.tif]

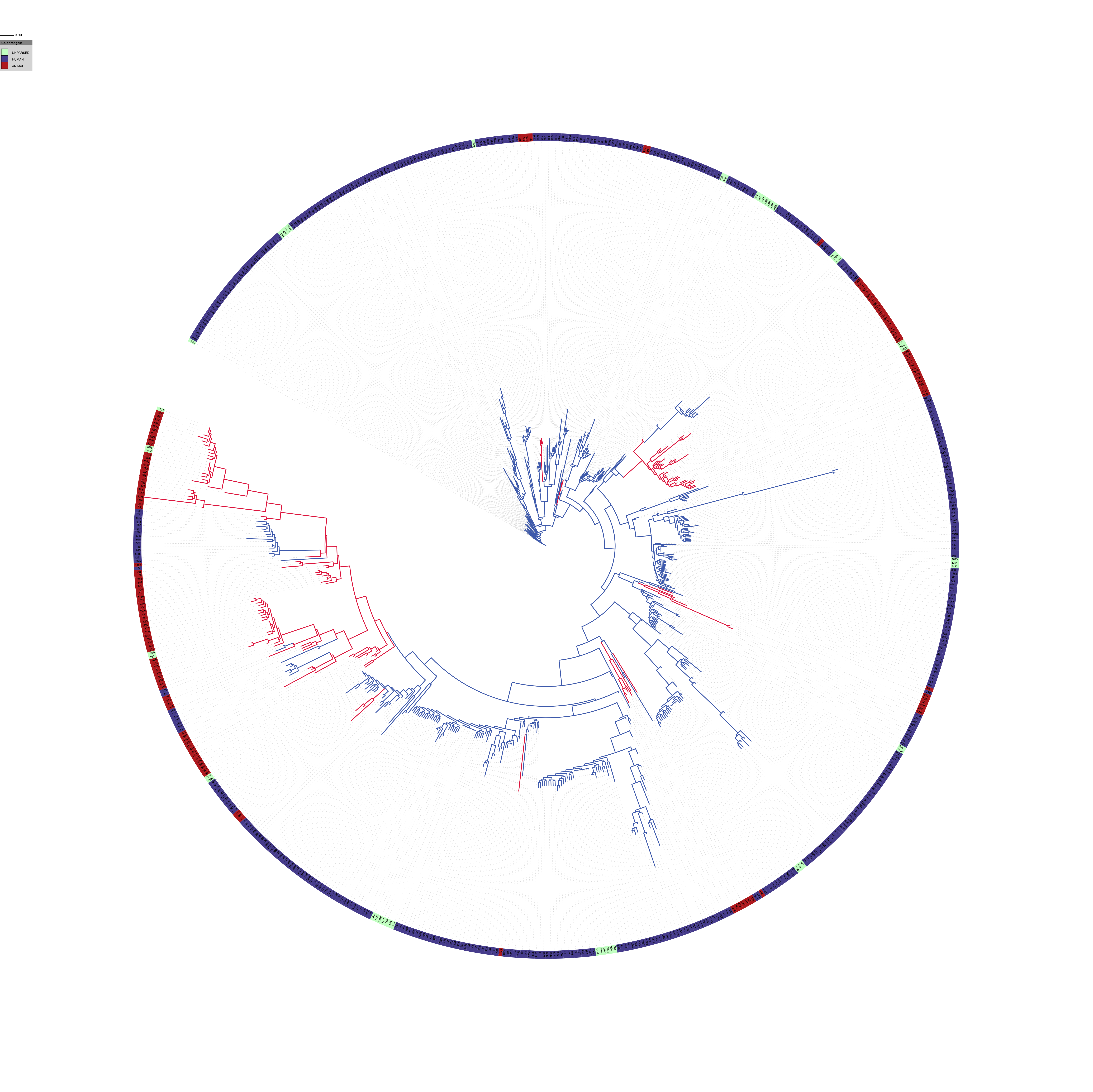

Supplement: Figure S5 — A maximum likelihood phylogeny of 696 MLST STs built using 6/7 MLST genes, excluding glpf . A maximum likelihood phylogeny of 696 MLST STs derived from human and animal hosts. Branch colours describe habitat associations inferred by AdaptML (Human – Blue, Animal – Red). The colours of the tip labels describe the input host assignment for each sequence type, red for animal, blue for human. Tip labels coloured green represent STs that formed polytomies as a result of the glpfc gene being excluded and were unparsed by the algorithm. (TIF) [file pone.0062369.s005.tif]

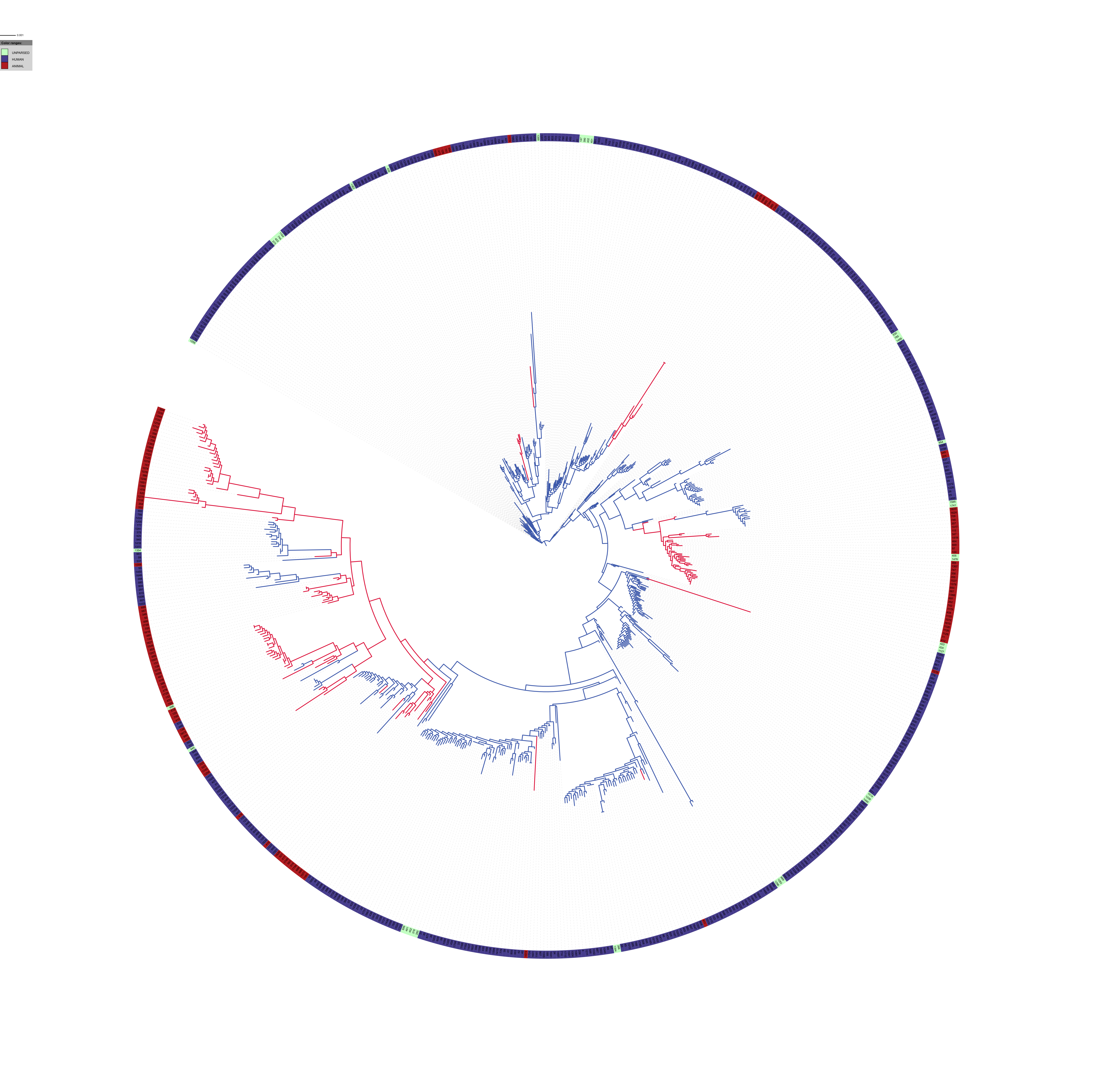

Supplement: Figure S6 — A maximum likelihood phylogeny of 696 MLST STs built using 6/7 MLST genes, excluding gmk_ . A maximum likelihood phylogeny of 696 MLST STs derived from human and animal hosts. Branch colours describe habitat associations inferred by AdaptML (Human – Blue, Animal – Red). The colours of the tip labels describe the input host assignment for each sequence type, red for animal, blue for human. Tip labels coloured green represent STs that formed polytomies as a result of the gmk_ gene being excluded and were unparsed by the algorithm. (TIF) [file pone.0062369.s006.tif]

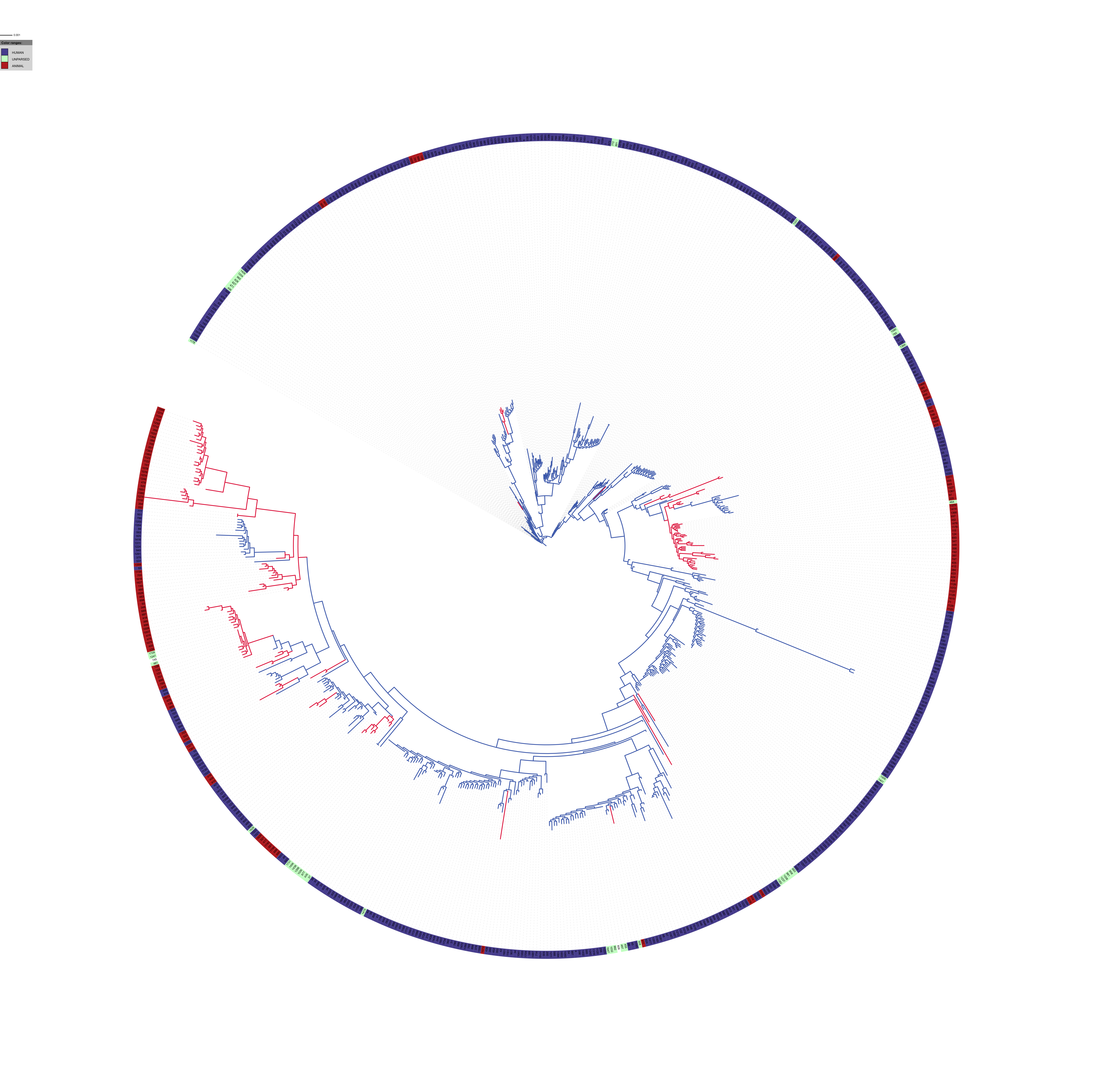

Supplement: Figure S7 — A maximum likelihood phylogeny of 696 MLST STs built using 6/7 MLST genes, excluding pta_ . A maximum likelihood phylogeny of 696 MLST STs derived from human and animal hosts. Branch colours describe habitat associations inferred by AdaptML (Human – Blue, Animal – Red). The colours of the tip labels describe the input host assignment for each sequence type, red for animal, blue for human. Tip labels coloured green represent STs that formed polytomies as a result of the pta_ gene being excluded and were unparsed by the algorithm. (TIF) [file pone.0062369.s007.tif]

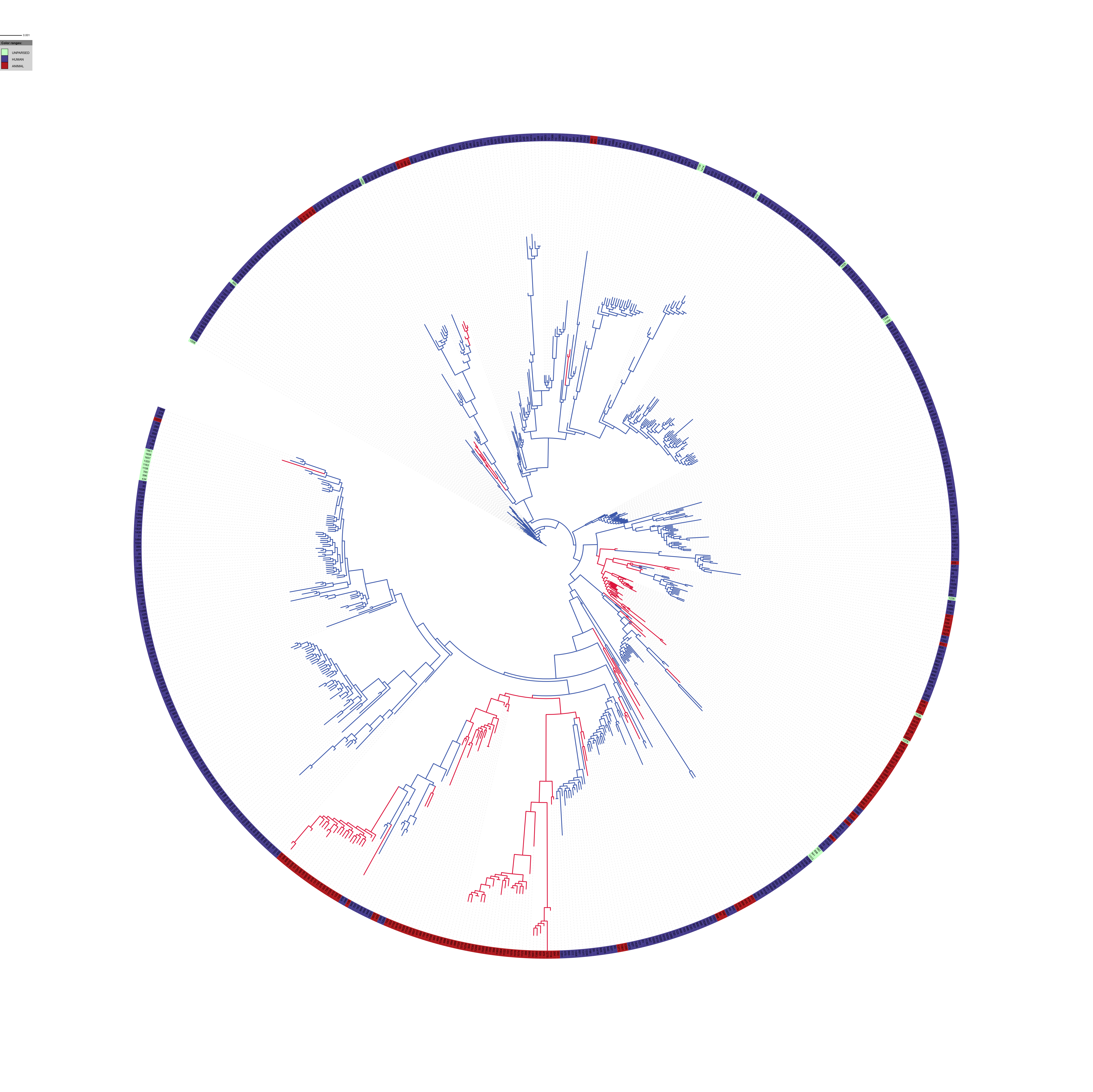

Supplement: Figure S8 — A maximum likelihood phylogeny of 696 MLST STs built using 6/7 MLST genes, excluding tpi_ . A maximum likelihood phylogeny of 696 MLST STs derived from human and animal hosts. Branch colours describe habitat associations inferred by AdaptML (Human – Blue, Animal – Red). The colours of the tip labels describe the input host assignment for each sequence type, red for animal, blue for human. Tip labels coloured green represent STs that formed polytomies as a result of the tpi_ gene being excluded and were unparsed by the algorithm. (TIF) [file pone.0062369.s008.tif]

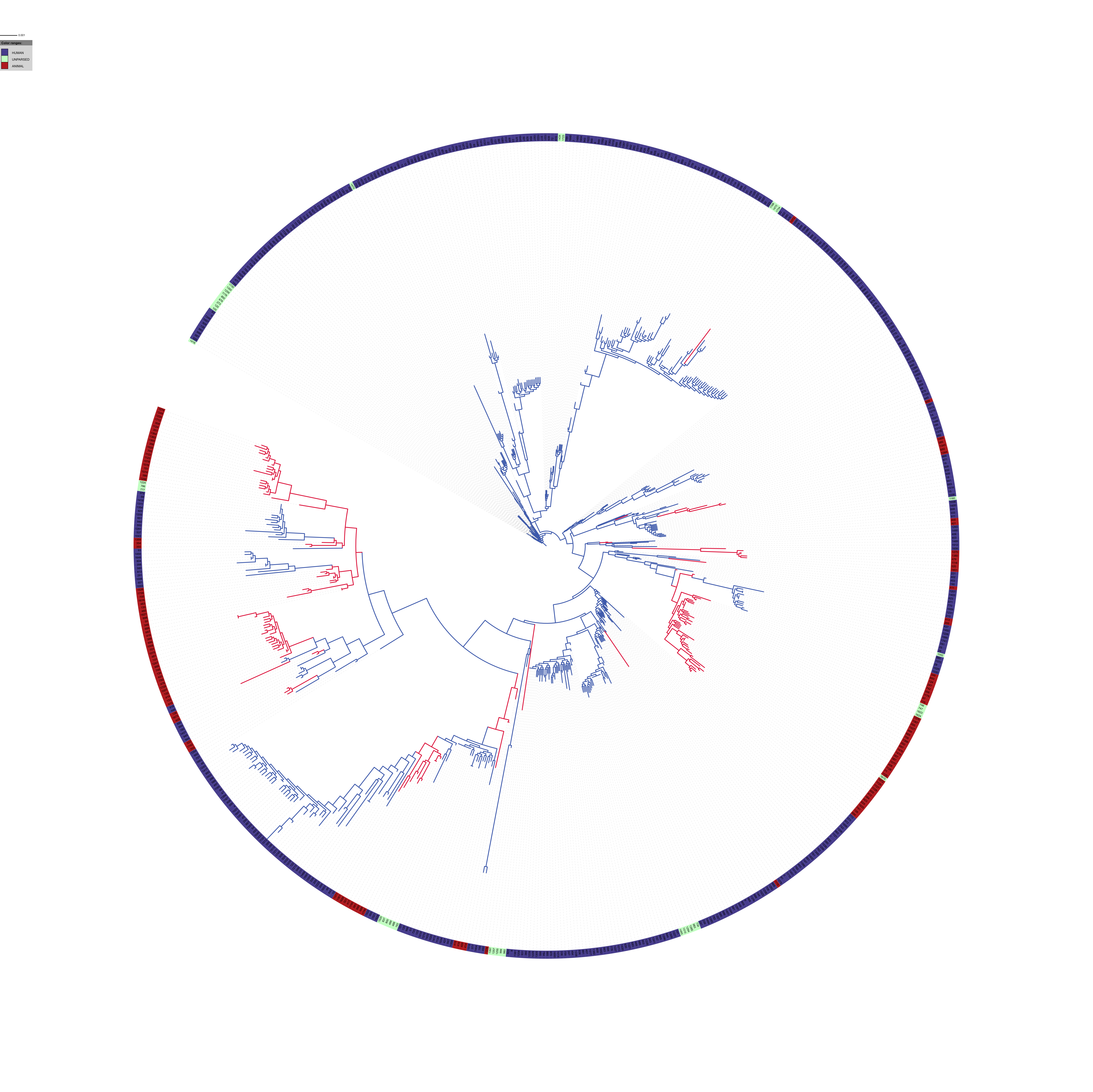

Supplement: Figure S9 — A maximum likelihood phylogeny of 696 MLST STs built using 6/7 MLST genes, excluding yqil . A maximum likelihood phylogeny of 696 MLST STs derived from human and animal hosts. Branch colours describe habitat associations inferred by AdaptML (Human – Blue, Animal – Red). The colours of the tip labels describe the input host assignment for each sequence type, red for animal, blue for human. Tip labels coloured green represent STs that formed polytomies as a result of the yqil gene being excluded and were unparsed by the algorithm. (TIF) [file pone.0062369.s009.tif]
