# Supplementary material for: Historical Zoonoses and Other Changes in Host Tropism of Staphylococcus aureus, Identified by Phylogenetic Analysis of a Population Dataset
Source: PLoS One. 2013 May 7;8(5):e62369. doi: 10.1371/journal.pone.0062369 (PMC3647051; doi:10.1371/journal.pone.0062369)
Supplement: Table S1 — Total numbers of isolates for each species, for all STs, within clades arising from a host switching event. Total numbers of isolates for each species, for all STs, within clades arising from a host switching event; these are sorted into animal clades arising from a zoonosis and human clades arising from an anthroponosis. (DOCX) [file pone.0062369.s010.docx]

| **Clade** | **Species** | | | | | | | |
| --- | --- | --- | --- | --- | --- | --- | --- | --- |
|  | **Human** | **Cow** | **Sheep** | **Pig** | **Goat** | **Chicken** | **Rabbit** | **Total** |
|  | **Animal** | | | | | | | |
| **136** |  | 2 |  |  |  |  |  | 2 |
| **411** |  | 1 |  |  |  |  |  | 1 |
| **414** |  |  |  |  |  |  | 1 | 1 |
| **1073** |  | 1 |  |  |  |  |  | 1 |
| **1119** |  | 1 |  |  |  |  |  | 1 |
| **1276** |  | 1 |  |  |  |  |  | 1 |
| **400** |  | 2 |  |  |  |  |  | 2 |
| **425** |  | 1 | 2 |  |  |  |  | 3 |
| **1361** |  | 2 |  |  |  |  |  | 2 |
| **409** |  |  |  |  |  |  | 3 | 3 |
| **522** |  |  | 2 |  | 7 |  |  | 9 |
| **385** | 1 | 1 |  | 1 |  | 4 |  | 7 |
| **126** |  | 187 |  | 2 | 5 |  |  | 194 |
| **130** | 1 | 5 | 11 |  | 17 |  |  | 34 |
| **133** |  | 50 | 11 |  | 45 |  |  | 106 |
| **151** |  | 251 | 1 |  | 1 |  |  | 253 |
| **97** | 20 | 332 | 1 | 7 | 2 |  | 2 | 364 |
|  | **Human** | | | | | | | |
| **25** | 50 | 78 |  | 1 | 2 |  |  | 131 |
| **59** | 40 | 1 |  |  |  |  |  | 41 |
| **93** | 6 |  |  |  |  |  |  | 6 |
