# Supplementary material for: Historical Zoonoses and Other Changes in Host Tropism of Staphylococcus aureus, Identified by Phylogenetic Analysis of a Population Dataset
Source: PLoS One. 2013 May 7;8(5):e62369. doi: 10.1371/journal.pone.0062369 (PMC3647051; doi:10.1371/journal.pone.0062369)
Supplement: Table S2 — Table listing the total numbers of STs and isolates for each clade arising from a host-switching event. STs and isolates are subdivided into those of either human (H) or animal (A) origin. (DOCX) [file pone.0062369.s011.docx]

| **Clade** | **STs** | | | **Isolates** | | |
| --- | --- | --- | --- | --- | --- | --- |
|  | H | A | Total | H | A | Total |
| **Animal** | |  |  |  |  |  |
| **136** | 0 | 1 | 1 | 0 | 1 | 1 |
| **411** | 0 | 1 | 1 | 0 | 1 | 1 |
| **414** | 0 | 1 | 1 | 0 | 1 | 1 |
| **1073** | 0 | 1 | 1 | 0 | 1 | 1 |
| **1119** | 0 | 1 | 1 | 0 | 1 | 1 |
| **1276** | 0 | 1 | 1 | 0 | 1 | 1 |
| **400** | 0 | 2 | 2 | 0 | 2 | 2 |
| **425** | 0 | 2 | 2 | 0 | 3 | 3 |
| **1361** | 0 | 2 | 2 | 0 | 2 | 2 |
| **409** | 0 | 3 | 3 | 0 | 3 | 3 |
| **522** | 0 | 3 | 3 | 0 | 9 | 9 |
| **385** | 1 | 5 | 6 | 1 | 6 | 7 |
| **126** | 0 | 9 | 9 | 0 | 194 | 194 |
| **130** | 1 | 15 | 16 | 1 | 33 | 34 |
| **133** | 0 | 21 | 21 | 0 | 106 | 106 |
| **151** | 0 | 29 | 29 | 0 | 253 | 253 |
| **97** | 4 | 32 | 36 | 20 | 344 | 364 |
| **Human** |  |  |  |  |  |  |
| **25** | 10 | 4 | 14 | 50 | 81 | 131 |
| **59** | 15 | 0 | 15 | 40 | 1 | 41 |
| **93** | 0 | 1 | 1 | 7 | 0 | 7 |
