# Supplementary material for: Historical Zoonoses and Other Changes in Host Tropism of Staphylococcus aureus, Identified by Phylogenetic Analysis of a Population Dataset
Source: PLoS One. 2013 May 7;8(5):e62369. doi: 10.1371/journal.pone.0062369 (PMC3647051; doi:10.1371/journal.pone.0062369)
Supplement: Table S3 — List of isolates in the CC25 clade, showing correction for sampling. This table lists the specific number of isolates from each host species for all STs found in the CC25 clade. The uncorrected numbers represent the totals as listed in the database, while the sample-corrected numbers represent the totals if only a single isolate of any given ST is included per species for each unique study listed in the database. (DOCX) [file pone.0062369.s012.docx]

|  | Uncorrected | | | | Sample-corrected | | | |
| --- | --- | --- | --- | --- | --- | --- | --- | --- |
| ST | Human | Cow | Pig | Goat | Human | Cow | Pig | Goat |
| 25 | 39 | 74 | 1 | 1 | 5 | 4 | 1 | 1 |
| 26 | 1 | 3 |  |  | 1 | 2 |  |  |
| 28 | 1 |  |  |  | 1 |  |  |  |
| 437 | 1 |  |  |  | 1 |  |  |  |
| 781 | 1 |  |  |  | 1 |  |  |  |
| 880 | 1 |  |  |  | 1 |  |  |  |
| 1029 | 1 |  |  |  | 1 |  |  |  |
| 1030 | 1 |  |  |  | 1 |  |  |  |
| 1042 | 1 |  |  |  | 1 |  |  |  |
| 1226 | 1 |  |  |  | 1 |  |  |  |
| 1372 |  | 1 |  |  |  | 1 |  |  |
| 1492 | 1 |  |  |  | 1 |  |  |  |
| 1595 | 1 |  |  |  | 1 |  |  |  |
| 1741 |  |  |  | 1 |  |  |  | 1 |
| Total | 50 | 78 | 1 | 2 | 16 | 7 | 1 | 2 |
