# Supplementary material for: Historical Zoonoses and Other Changes in Host Tropism of Staphylococcus aureus, Identified by Phylogenetic Analysis of a Population Dataset
Source: PLoS One. 2013 May 7;8(5):e62369. doi: 10.1371/journal.pone.0062369 (PMC3647051; doi:10.1371/journal.pone.0062369)
Supplement: Table S4 — Pairwise similarity between the topologies of all seven jackknife trees and the main seven-gene phylogeny. Proportions of similarity between the topologies of the main tree (figure 1) and the jackknife trees built from 6/7 MLST genes (figures S3, S4, S5, S6, S7, S8, S9). Proportions represent the number of all nodes that are identical in both trees. (DOCX) [file pone.0062369.s013.docx]

|  | **MAIN** | **-arcc** | **-aroe** | **-glpf** | **-gmk_** | **-pta_** | **-tpi_** | **Yqil** |
| --- | --- | --- | --- | --- | --- | --- | --- | --- |
| **MAIN** | 1 | 0.2609 | 0.2277 | 0.3271 | 0.2061 | 0.2837 | 0.2616 | 0.2596 |
| **-arcc** |  | 1 | 0.1938 | 0.2450 | 0.2031 | 0.2193 | 0.1928 | 0.2442 |
| **-aroe** |  |  | 1 | 0.2155 | 0.1740 | 0.1933 | 0.1883 | 0.1859 |
| **-glpf** |  |  |  | 1 | 0.2061 | 0.2745 | 0.2392 | 0.2197 |
| **-gmk_** |  |  |  |  | 1 | 0.2055 | 0.1689 | 0.1797 |
| **-pta_** |  |  |  |  |  | 1 | 0.2257 | 0.2227 |
| **-tpi_** |  |  |  |  |  |  | 1 | 0.2135 |
| **yqil** |  |  |  |  |  |  |  | 1 |
