# Supplementary material for: Historical Zoonoses and Other Changes in Host Tropism of Staphylococcus aureus, Identified by Phylogenetic Analysis of a Population Dataset
Source: PLoS One. 2013 May 7;8(5):e62369. doi: 10.1371/journal.pone.0062369 (PMC3647051; doi:10.1371/journal.pone.0062369)
Supplement: Table S5 — Comparison of the number of inferred host switches in seven-gene, and six-gene jackknife phylogenies. A list describing the basal habitat in both the main tree (figure 1) and the seven jackknife trees (figures S3, S4, S5, S6, S7, S8, S9), as well as the numbers of zoonoses and anthroponoses observed in each. (DOCX) [file pone.0062369.s014.docx]

| **Tree** | **Basal Habitat** | **Zoonoses** | **Anthroponoses** | **Total** |
| --- | --- | --- | --- | --- |
| **MAIN** | Human | 3 | 17 | 20 |
| **-arcc** | Human | 9 | 11 | 20 |
| **-aroe** | Human | 1 | 14 | 15 |
| **-glpf** | Human | 6 | 10 | 16 |
| **-gmk_** | Human | 8 | 10 | 18 |
| **-pta_** | Human | 3 | 17 | 20 |
| **-tpi_** | Human | 6 | 16 | 22 |
| **-yqil** | Human | 6 | 17 | 23 |
