# Supplementary material for: Historical Zoonoses and Other Changes in Host Tropism of Staphylococcus aureus, Identified by Phylogenetic Analysis of a Population Dataset
Source: PLoS One. 2013 May 7;8(5):e62369. doi: 10.1371/journal.pone.0062369 (PMC3647051; doi:10.1371/journal.pone.0062369)
Supplement: Table S6 — Dates of zoonotic habitat transitions. Dates of zoonotic habitat transitions in Figure 1, estimated using BEAST software with clock rate set to (3.3×10^−6^ substitutions per site per year). Date ranges represent the TMRCA (given by the treemodel.RootHeight parameter in BEAST) for the nodes at either end of a branch where the basal node is associated with the inferred animal habitat, and the distal node is associated with the inferred human habitat. Only the transitions giving rise to CC25 and CC59 had sufficient sequence information to generate a credible date. (DOCX) [file pone.0062369.s015.docx]

| **Clade** | **Geometric Mean** | **Median** |
| --- | --- | --- |
| **CC25** | 597.7 – 1095.3 | 507.4 – 975.6 |
| **CC59** | 538.5 – 987.1 | 471.7 – 866.1 |
