# Supplementary material for: Historical Zoonoses and Other Changes in Host Tropism of Staphylococcus aureus, Identified by Phylogenetic Analysis of a Population Dataset
Source: PLoS One. 2013 May 7;8(5):e62369. doi: 10.1371/journal.pone.0062369 (PMC3647051; doi:10.1371/journal.pone.0062369)
Supplement: Table S7 — List of bootstrap values for branches where switches occur. Bootstrap values of branches where host switches occur. Switches involving only a single ST are marked with an asterisk, and the bootstrap value of the immediate basal branch is given as a reference. (DOCX) [file pone.0062369.s016.docx]

| **Switch** | **Bootstrap Value** |
| --- | --- |
| **Anthroponoses** |  |
| **1073 *** | 57 % |
| **1276*** | 98 % |
| **400** | 96 % |
| **425** | 85 % |
| **1361** | 97 % |
| **409** | 91 % |
| **522** | 93 % |
| **385** | 95 % |
| **126** | 93 % |
| **130** | 100 % |
| **133** | 97 % |
| **151** | 74 % |
| **97** | 85 % |
| **Zoonoses** |  |
| **25** | 99 % |
| **59** | 87 % |
