# Supplementary material for: Historical Zoonoses and Other Changes in Host Tropism of Staphylococcus aureus, Identified by Phylogenetic Analysis of a Population Dataset
Source: PLoS One. 2013 May 7;8(5):e62369. doi: 10.1371/journal.pone.0062369 (PMC3647051; doi:10.1371/journal.pone.0062369)
Supplement: Table S8 — List of supplemental isolates. A table of supplementary animal isolates provided by EF which were included in the final dataset along with the isolates already present within the S. aureus MLST database. (DOCX) [file pone.0062369.s017.docx]

| **Host Species** | **Country of Origin** | **ST** |
| --- | --- | --- |
| **Pig** | France | 9 |
| **Goat** | France | 15 |
| **Pig** | France | 30 |
| **Pig** | France | 30 |
| **Cow** | France | 71 |
| **Cow** | France | 71 |
| **Cow** | France | 97 |
| **Cow** | France | 97 |
| **Cow** | France | 97 |
| **Cow** | France | 97 |
| **Cow** | France | 97 |
| **Chicken** | France | 221 |
| **Pig** | France | 378 |
| **Cow** | France | 384 |
| **Pig** | France | 385 |
| **Chicken** | France | 385 |
| **Goat** | France | 386 |
| **Pig** | France | 387 |
| **Sheep** | France | 388 |
| **Cow** | France | 389 |
| **Cow** | France | 389 |
| **Cow** | France | 400 |
| **Cow** | France | 401 |
| **Pig** | France | 402 |
| **Chicken** | France | 403 |
| **Chicken** | France | 404 |
| **Pig** | France | 405 |
| **Pig** | France | 406 |
| **Pig** | France | 406 |
| **Rabbit** | France | 407 |
| **Cow** | France | 408 |
| **Rabbit** | France | 409 |
| **Goat** | France | 410 |
| **Cow** | France | 411 |
| **Cow** | France | 412 |
| **Cow** | France | 413 |
| **Rabbit** | France | 414 |
| **Rabbit** | France | 415 |
| **Rabbit** | France | 416 |
| **Sheep** | France | 417 |
| **Sheep** | France | 418 |
| **Sheep** | France | 419 |
| **Goat** | France | 420 |
| **Rabbit** | France | 421 |
| **Cow** | France | 423 |
| **Cow** | France | 423 |
| **Cow** | France | 424 |
| **Sheep** | France | 425 |
